# From traditional musicians to digital musicians: a study on talent transformation in the music industries driven by AI technology

**DOI:** 10.3389/fsoc.2026.1852712

**Published:** 2026-07-22

**Authors:** Wei Wang

**Affiliations:** School of Music, Hanshan Normal University, Chaozhou, China

**Keywords:** artificial intelligence, music industry, skill transformation, sustainable development, workforce development strategy

## Abstract

**Introduction:**

The rapid integration of artificial intelligence (AI) into the music industries is fundamentally reshaping occupational roles, skill requirements, and career pathways, posing critical challenges for workforce development. This transformation compels a shift from conventional, craftbased music-making toward digitally enabled, interdisciplinary practices.

**Methods:**

Employing a phenomenological qualitative research design, this study conducted in-depth, semistructured interviews with 20 stakeholders across four key groups-government officials, higher education faculty, enterprise managers, and frontline musicians- within the Beijing music industry. Data were analyzed using a six-step thematic analysis process facilitated by NVivo 14.

**Results:**

The findings reveal that AI is systematically permeating music creation, production, distribution, and copyright management, driving a shift toward more digital, collaborative, and data-informed roles. However, significant cognitive gaps and divergent adaptive capacities among stakeholder groups have created structural imbalances within the talent development ecosystem.

**Discussion:**

To address these challenges, this paper proposes an empirically grounded, four-pillar workforce development framework comprising institutional support, educational reform, enterprise engagement, and community development, emphasizing systemic coordination to build a resilient digital music talent ecosystem. This study advances theoretical understanding of labor market transformation in creative industries and provides practical pathways for regions seeking to foster a sustainable and inclusive music workforce in the age of AI.

## 1 Introduction

With the rapid advancement of artificial intelligence (AI) technology, the music industries are undergoing profound technological and organizational changes (Elberse, 2024; Wikström, 2020). Generative AI (AIGC), sound synthesis technology, intelligent mixing algorithms, and big data-based copyright distribution systems are being

systematically integrated into the entire lifecycle of the music industry chain, leading to structural reorganization from creation and production to distribution and management (Carnovalini and Rodà, 2020; Huang et al., 2024). These technologies are redefining methods of music creation and production alongside the skills required, making workforce restructuring and career transformation within the music industries one of the most urgent challenges today (Hesmondhalgh and Meier, 2018; Prior, 2020).

While digitization has been an ongoing process in music since at least the 1980s (Théberge, 2021), the recent wave of machine learning and generative AI represents a qualitative shift. For this paper, “traditional musicians” refers to practitioners—such as session performers, recording engineers, and classically trained composers—whose primary working methods are rooted in acoustic instrumental proficiency, analog or basic digital recording techniques, and craft-based, experience-driven decision-making. In contrast, “digital musicians” are defined as practitioners who possess a hybrid skillset integrating professional musical competency with digital literacy, data interpretation, human-computer interaction, and interdisciplinary collaboration skills necessary to work effectively with intelligent systems (Baym, 2018; Prior, 2020; Rogers, 2023).

Among all music professional groups, these traditional musicians (such as composers, performers, and recording engineers) are particularly directly impacted by AI technology (Born and Devine, 2015; Webster, 2024). The algorithms, sound perception, and handicraft processes that they have long relied on are being redefined by algorithmic models, automated systems, and digital platforms (Briot et al., 2020; Ji et al., 2021). Certain conventional roles are even exhibiting a tendency of being taken over or re-defined (Frey and Osborne, 2017; Sturm, 2022). Conversely, multifaceted digital musicians—who have professional musical talent, digital literacy, interdisciplinary knowledge, and the ability to cooperate with intelligent systems—are experiencing increased demand in the global market, though supply remains critically low (Baym, 2018; Bennett and Hennekam, 2018). Thus, the shift of traditional musicians to digital musicians is considered one of the key ways to secure the sustainable evolution of the workforce of the music industries (Rogers, 2023; Théberge, 2021).

In a larger context, AI technology is transforming the labor organization of the global creative sector, and it has a definite skill-biased impact (Acemoglu and Restrepo, 2020; Autor, 2022). The music industries in developing nations are often characterized by a structural paradox of rapid technological changes and a lack of relevant skills in the market (De Beukelaer and Spence, 2021; Wang and Zhu, 2021). The music sector, as such, is highly dependent on creativity, involves complicated copyright systems, and requires multi-role interaction, making it much more difficult to transform than general manufacturing or standardized service industries (Hesmondhalgh and Baker, 2021; Williamson and Cloonan, 2016). According to available studies, technological advancements are frequently characterized by increased skill gaps and job insecurity, which in turn exacerbate career anxiety in practitioners (Comunian et al., 2020; Vaag et al., 2014).

Nonetheless, the current system of music education and career growth opportunities has not been responding to the AI-related challenge of skill transformation adequately so far, including in such aspects as the development of digital, multifaceted music talents (Bennett, 2019; Gaunt and Westerlund, 2021). The music industry is a major cultural pillar and also a component of the digital creative economy, particularly in cultural hub cities of China, including Beijing (Keane and Zhang, 2022; Kong, 2020). In this regard, the systematic shift of traditional musicians to digital musicians has considerable practical value regarding the further evolution of the music industry toward high-quality levels and its competitiveness on the international scale (Flew and Cunningham, 2021; UNESCO, 2022).

This research takes the Beijing music industry as a case study whereby it seeks to analyze the changing nature of AI technology in altering skill structures and job requirements, how major stakeholders view change, and how talent development strategies can be used to facilitate this change. The paper addresses the following two research questions (RQs):

RQ1: how does AI technology reshape job configurations and skill structures in the music industries, and how do key stakeholders perceive these changes?
RQ2: how should music workforce development strategies be optimized to facilitate an effective transition from traditional musicians to digital musicians?

## 2 Literature review

### 2.1 Music industry workforce evolution amid AI-driven digital transformation

AI-assisted composition systems, automated music production systems, intelligent distribution and recommendation systems, and other artificial intelligence technologies have quickly infiltrated key stages of the music creation, production, and distribution process (Carnovalini and Rodà, 2020; Huang et al., 2024). This technology penetration drives a mass movement out of conventional artisanal modes of operation based on traditional methods toward digital and algorithm-enhanced practices in the music industries (Ji et al., 2021; Briot et al., 2020). The role of AI-generated music production, intelligent production processes, and digital rights management systems in redefining creative efficiency, revenue models, and organizational structures has been the subject of a significant amount of research (Elberse, 2024; Wikström, 2020; Prey, 2020).

At the same time, this technological transformation also basically alters the ecosystem of skills resources and the structure of professional jobs in the music industries (Born and Devine, 2015; Théberge, 2021). Evidence from studies investigating the effect of automation on creative jobs indicates that, in the early stages of development, people lose jobs that include repetitive and routine duties (Frey and Osborne, 2017; Autor, 2022). Within the music industries, this is transforming into systemic restructuring, which includes the redefinition of professional roles of session musicians and sound engineers into creative directors and data-driven A&R experts (Webster, 2024; Arditi, 2021). For instance, the role of the traditional recording engineer, once defined by hands-on operation of mixing desks and outboard gear, is increasingly giving way to the “AI-assisted producer” who uses intelligent algorithms for mixing and mastering, shifting the core competency from manual dexterity to curatorial and technical oversight.

The integration of AI in music can be understood through a heuristic model of three interconnected shifts in capability requirements. This is a conceptual framework derived from synthesizing the literature and the empirical findings of this study. The first shift is the automation of technical processes: routine tasks like basic mixing, mastering, arrangement, and even audio repair are increasingly handled by AI, freeing practitioners from repetitive labor but also devaluing these craft skills (Sturm, 2022; Collins et al., 2020). The second shift involves the augmentation of creative and managerial roles: AI becomes a collaborative partner, assisting with idea generation, providing data-driven audience insights, and informing strategic decisions, thus requiring practitioners to develop skills in human-AI co-creation and data interpretation (Carnovalini and Rodà, 2020; Herremans et al., 2017). The third shift is the emergence of entirely new roles and competency clusters, where musical practitioners need to evolve beyond conventional instrumental or singing skills into professional technologies, information processing, and platform operations, developing adaptive creative problem-solving abilities and becoming what can be described as digital musicians or intelligent musical talent (Baym, 2018; Prior, 2020). This conceptual model helps move the discussion beyond a simple “automation threat” narrative to a more nuanced understanding of co-evolution between human practitioners and intelligent systems.

In terms of labor economics, the music industries pass through a stage of skill transformation associated with coexisting substitutive automation (AI replacing routine production functions) and augmentative collaboration (AI expanding creative abilities) (Acemoglu and Restrepo, 2020; Tschang and Mezquita, 2021). This two-way dynamic gives both chances for creative growth and workforce adaptation challenges (Leyshon, 2020; Marshall, 2015).

Indeed, AI-driven skill structure transformation has shifted workforce development from traditional conservatory-centered single-supply models toward multi-stakeholder collaborative governance systems, as individual entities cannot independently address systematic restructuring challenges (Bennett and Hennekam, 2018; Burnard et al., 2020). Governments can serve as architects of institutions, working on workforce development by providing directions on cultural policy, introducing changes to the copyright framework, and providing subsidies to creative industries (Flew, 2021; Kong, 2020). Music businesses and platforms are not only technology drivers but also shapers of employment ecosystems that are critical in position restructuring, definition of skills needed, and opportunities for professional development (Hracs, 2015; Prey, 2020). Talent pipeline development and knowledge system construction are responsibilities of educational institutions, which are all the more likely to become forces for curriculum reform, the establishment of interdisciplinary competency frameworks involving music and technology, and the deepening of ties with the industry (Bennett, 2019; Bartleet and Higgins, 2018).

International bodies and major music markets have come up with several workforce development strategies with training and re-education at their core to overcome the issue of skill transformation. UNESCO focuses on developing lifelong learning systems and creative capacity building systems of the cultural and creative sectors (UNESCO, 2022). The European Music Council promotes digital expertise standardization and cross-national professional growth with the assistance of collaborative frameworks (European Music Council, 2021). The integration of industry and education and collaboration of platforms and institutions in cultural creative areas have been given priority by China, creating, over time, a multipolar system of talent cultivation that is led by government cultural policy, involves the participation of enterprise platforms, and modernizes the conservatoires (Keane and Zhang, 2022; Wang and Zhu, 2021).

However, experience shows that in the music industries, tripartite coordination systems between government, industry, and educational institutions are fraught with long-standing struggles such as the heterogeneity of goals (artistic and commercial), the lack of resources (between old and new institutions), and the lack of time (slow learning processes in educational institutions and rapid changes in technology) (Tagg, 2012; Burnard et al., 2020). These problems are causing gaps between music education, labor market realities, and technological requirements in workforce transformation systems, creating institutional coordination challenges and gaps in strategies (Beeching, 2010; Vaag et al., 2014).

Music conservatories and universities are characterized by a lack of responsiveness to technological change and an inability to adjust curricula to professional needs, contributing to the dispersion of goals and a lack of adequate capabilities in relation to workforce development (Gaunt and Westerlund, 2021; King and Waddington-Jones, 2011). Conventional paradigms of music education that focus on mastery of performance and theoretical knowledge typically overlook digital literacy, entrepreneurial abilities, and technological literacy, which are becoming increasingly vital to career sustainability (Smilde, 2009; Bennett and Stanberg, 2006).

Competency profiling and skill standard systems for digital music professionals, and approaches for talent development, have primarily examined conceptual frameworks and industry trend analyses (Webster, 2024; Dumlavwalla, 2020). This work still does not adequately provide empirical analysis from experienced music practitioners such as working musicians, educators, producers, platform managers, and industry executives, and ignores their perspectives on technological changes, transformation processes, and influencing factors such as psychological barriers, organizational support structures, and adaptive learning challenges during skill transitions (Hesmondhalgh and Baker, 2021; Comunian et al., 2020). The literature consistently removes agency dimensions within these transformation processes, creating substantial space for inquiry and theory development in future research initiatives (McRobbie, 2016; Hennekam and Bennett, 2017).

### 2.2 Theoretical foundations of skill reconstruction in the music industry

The music industries are essentially labor-intensive creative industries, which have conventionally depended on a system of developing skills through apprenticeship and reflected artistry (Throsby and Zednik, 2011; McRobbie, 2016). Structural problems of skill incompatibilities and talent deficits in new digital jobs, and backward professional development regimes due to increased technological complexity, convergence of platforms, and changing consumption patterns have been formed (Hesmondhalgh and Baker, 2013; Williamson and Cloonan, 2016). These concerns are especially intensive in changing music markets where the absence of a technological infrastructure, weak knowledge transfer systems, and ineffective industry-education connections are noticeable (De Beukelaer and Spence, 2019; Kong, 2020).

These workforce challenges have been aggravated by population changes, shifts in career ambitions among young people, and the streaming economy (Marshall, 2015; Prey, 2020). These competency gaps have been identified by many regions as huge bottlenecks to the development of sustainable music industries and the cultural creative economy (Kong, 2020; UNESCO, 2022).

These complex people management problems require strong theoretical models to learn how change happens. Even though the concept of human capital theory underlines that professional value is based on educational and skill investment (Becker, 1964; Throsby, 1999), it is not able to entirely explain the changes in creative abilities or role changes in the music industries as influenced by AI technologies (Hesmondhalgh and Meier, 2018). To address this theoretical gap, dynamic capabilities theory (Teece et al., 1997; Teece, 2007) and organizational learning theory (Argyris and Schön, 1978; Senge, 1990) explain how creative organizations develop exploratory learning and capability reconstruction during technological transformations (March, 1991; Crossan et al., 1999).

Skill polarization (Autor et al., 2003; Goos et al., 2014) is another concept that forecasts that as technology advances, the number of middle-skill creative jobs continues to decline, and the number of high-cognitive creative jobs and platform-mediated opportunities rises (Acemoglu and Restrepo, 2020; Autor, 2022). These theories, all together, become macro-explanatory frameworks for comprehending music organization perception and action regarding technological change, adaptation of professional structures, and involvement of creative workers (Hesmondhalgh and Meier, 2018; Born and Devine, 2015).

According to such frameworks, viewpoints in organizational learning shed light on adaptation processes during change. Organizational learning theory stresses that the renewal of knowledge is based on reflective practice of the change (Nonaka and Takeuchi, 1995; Argote and Miron-Spektor, 2011), and the theory of ambidextrous learning suggests that organizations should strike a balance between the exploitation of past artistic experience and the discovery of new technological possibilities (March, 1991; O'Reilly and Tushman, 2013). In music industry contexts, these frameworks are specific to the case of how music organizations, platforms, and individual artists react to external AI disruption and remain creatively authentic while continuing to develop talent systems (Leyshon, 2020; Hracs, 2015). In addition, they also offer theoretical underpinnings on the perceptions and adaptive responses of those stakeholders who represent government cultural agencies, music enterprises/platforms, institutions of higher learning, and musician constituencies (Flew, 2021; Bennett and Hennekam, 2018).

The idea above shows the changing priorities in the research of the music workforce. The pressures of fast-evolving AI technologies have shifted the research agenda from studying labor precarity to studying creative agency and skill transition potentials (Hesmondhalgh and Baker, 2013; Comunian et al., 2020). It suggests a strategic change in the focused approach toward traditional musicians to the development of digital musicians or intelligent musical talent that can creatively cooperate with humans (Prior, 2020; Baym, 2018).

Nevertheless, when considered in its entirety, current studies have shown that there are considerable constraints. The majority of music workforce development research focuses on individual dimensions separately, such as educational curriculum gaps (Bennett, 2019; Gaunt and Westerlund, 2021), new skills (Webster, 2024; Dumlavwalla, 2020), platform technological change (Prey, 2020; Arditi, 2021), and cultural policy frameworks (Flew, 2021; Wang and Zhu, 2021), without addressing the dynamic interaction and cognitive diversification across the various stakeholders. Most literature focuses on phenomenon description rather than enacting systematic integration of perspectives supporting the logical chain from AI technology drivers → skill structure transformation → multi-stakeholder responses → strategic system construction (Hesmondhalgh and Meier, 2018).

In order to address these shortcomings, institutional coordination and multi-governance theories (Powell and DiMaggio, 1991; Rhodes, 1997) offer methodical ways to make AI-oriented music workforce approaches (Ansell and Gash, 2008; Emerson et al., 2012). Workforce transformation processes need the involvement of various institutional actors during systemic change (Jessop, 2002; Kooiman, 2003). An analytical framework that consists of diverse theoretical views is meaningful to explain the transformation trajectory of creative work in the music sector (O'Connor and Gu, 2020; Flew and Cunningham, 2021).

These theoretical backgrounds form the basis of this study's analytical lens. Dynamic capabilities theory, organizational learning theory, and institutional coordination frameworks provide an understanding of how various stakeholder groups adapt to technological disruption through redefining creative roles, revising their knowledge systems, and refocusing their development strategies (Teece, 2007; Zollo and Winter, 2002). Collectively, these take on the perspectives of how major actors in the music industries perceive and react to workforce change in an AI-based world (Hesmondhalgh and Baker, 2021; Comunian et al., 2020).

## 3 Methodology

### 3.1 Research methodology

Current literature mainly uses quantitative design types, i.e., questionnaire surveys or the analysis of secondary data, as the main methods, providing a restricted amount of information about the lived experience or cognitive distinctions in the process of talent transformation (Hesse-Biber and Johnson, 2015; Pratt, 2020). To fill this gap, this paper discusses the phenomenon of transformation of the music industries in terms of creating digital musicians out of traditional musicians using AI technology—a complicated process that brings together changes in technology, organizational responses, and coordination of institutions (Flew and Cunningham, 2020; Webster, 2020).

Given the need to explore stakeholders' lived experiences and perceptions, this study adopts a phenomenological qualitative research approach (Creswell and Poth, 2018; Van Manen, 2016). This approach is especially appropriate to examine how participants perceive organizational change and skill change in their social and institutional surroundings (Gioia et al., 2013; Cunliffe, 2011). It allows one to understand the process of skill restructuring by participants and gain a depth understanding of how participants experience and create meaning in the process (Moustakas, 1994; Smith et al., 2021). This is particularly suited for uncovering how organizations and individuals locate, assimilate, and reconfigure capability resources concerning technological change (Eisenhardt et al., 2021; O'Reilly and Binns, 2019).

### 3.2 Research sampling

Beijing was selected as the research region for well-founded reasons: the music industry serves as a cultural pillar in the city, its population structure demonstrates regional representativeness, and in recent years, Beijing has actively promoted the digital transformation of its music industry, offering valuable case-study characteristics (Montgomery and Robinson, 2021; O'Connor and Gu, 2020). Sampling was conducted using purposive sampling techniques, with maximum variation sampling applied to ensure comprehensive perspectives and data saturation (Palinkas et al., 2015; Patton, 2015).

Based on recommendations from existing studies (Guest et al., 2006; Hennink et al., 2017), this research included 20 interview participants, covering four stakeholder categories. Government officials represent three departments: the Culture and Tourism Bureau (G1, G2), the Human Resources and Social Security Bureau (G3), and the Education Commission. Higher education representatives are professors from major music conservatoires and arts colleges in Beijing (E1–E4). Enterprise managers include a human resources manager, technical director, and project manager from major music production companies, independent music labels, and AI music technology businesses (M1–M5). Frontline musicians include composers, arrangers, recording engineers, young musicians, independent musicians, and female music creators (W1–W8). This sampling design guarantees coverage at the policy-making, education provision, organization response, and operation perception levels (Eisenhardt and Graebner, 2007; Yin, 2018).

It is important to note that the sample—with approximately five participants per stakeholder group—is best understood as an exploratory or scoping sample. It is appropriate for a qualitative study aiming for depth and theoretical insight rather than broad generalizability. The findings should be seen as identifying key themes and mechanisms for further investigation in more focused, sector-specific follow-up studies.

Table 1 provides the full demographic profile of the 20 respondents, who were government officials, university professors, corporate managers, and frontline musicians. There is a ratio of 60 percent males in the sample. Presentation of educational backgrounds is quite hierarchical, with government officials and university professors predominately possessing a master's or higher degree and frontline musicians predominantly with a bachelor's degree. Distribution of age and professional experience indicates apparent stratification (Lincoln and Guba, 1985; Morse, 2015), where government officials and university professors have an average age above 40 years and 15 years of professional experience. The frontline musicians are between 24 and 35 years old with an average tenure of about 6 years, whereas the corporate managers are around 37 with an average experience of 11–17 years.

**Table 1:** Demographic characteristics of respondents.

No.	ID	Category	Gender	Age	Education	Years of experience
01	G1	Government official	Male	43	Master's	18
02	G2	Government official	Female	40	Master's	15
03	G3	Government official	Male	45	Master's	20
04	E1	University professor	Male	48	Ph.D.	20
05	E2	University professor	Female	44	Ph.D.	16
06	E3	University professor	Male	42	Ph.D.	15
07	E4	University professor	Female	39	Master's	12
08	M1	Enterprise manager	Male	41	Master's	17
09	M2	Enterprise manager	Female	38	Master's	14
10	M3	Enterprise manager	Male	36	Bachelor's	13
11	M4	Enterprise manager	Female	34	Master's	11
12	M5	Enterprise manager	Male	37	Bachelor's	15
13	W1	Frontline musician	Male	35	Bachelor's	12
14	W2	Frontline musician	Male	32	Master's	10
15	W3	Frontline musician	Female	28	Bachelor's	6
16	W4	Frontline musician	Male	25	Bachelor's	3
17	W5	Frontline musician	Female	26	Bachelor's	4
18	W6	Frontline musician	Male	30	Master's	8
19	W7	Frontline musician	Female	24	Bachelor's	2
20	W8	Frontline musician	Male	27	Bachelor's	5

Out of 20 participants, 16 have direct experience in music projects using AI or digital technology. Projects involving corporate managers (M1–M5) and frontline musicians (W1–W8) have involved AI-assisted composition, intelligent mixing, digital distribution platforms, and copyright management systems, often using technology like AI composition tools, intelligent audio processing, digital audio workstations (DAWs), and streaming data analytics (Born and Devine, 2015; Prior, 2020). Moreover, the participants from higher education (E1–E4) have also interacted with AI applications by teaching and participating in the industry, such as AI-based music production tools, algorithmic composition, or smart systems to monitor copyright (Webster, 2020; Hesmondhalgh and Meier, 2018). Others were also involved in standard-setting and technical consultation in the area of music technology. This guarantees that the sample provides an informed, experience-based view among all the stakeholder groups, and hence multidimensional theoretical knowledge is gained (Gioia et al., 2013; Tracy, 2010; see Table 1).

### 3.3 Data collection

Semi-structured in-depth interviews were employed as the primary method for data collection (Brinkmann and Kvale, 2015; Kallio et al., 2016). Prior to the interviews, researchers contacted potential participants via telephone and email to confirm their willingness to participate and to schedule appointments (Jacob and Furgerson, 2012; Merriam and Tisdell, 2015). The interviews were conducted between September and December 2025, with each session lasting 40 to 70 min. With the participants' consent, the interviews were audio-recorded and subsequently transcribed for analysis (Barriball and While, 1994; Stuckey, 2013).

The interview guide was structured around three core research dimensions (Patton, 2015; Rubin and Rubin, 2012). The Impact Assessment dimension examined the influence of AI on the skill structure and job requirements within the music industries (Autor, 2022; Acemoglu and Restrepo, 2020). The Perception and Response Analysis dimension explored the cognitions and reactions of multiple stakeholders regarding talent transformation (Gioia et al., 2013; Weick et al., 2005). The Strategic Development dimension investigated the methods by which individuals and organizations facilitate the capability transformation of music talents and their continuous adaptation within an AI-driven environment (Teece, 2007; O'Reilly and Tushman, 2013).

Representative interview questions included: “In which specific segments or workflows of the music industries have you observed the primary application of AI? Can you provide concrete examples?” (Hesmondhalgh and Meier, 2018; Webster, 2020); “What measures has your organization, or organizations you are familiar with, taken to address the evolving skill requirements, and how effective have these initiatives been?” (Bennett and Hennekam, 2018; Comunian et al., 2020); and “To better respond to AI-driven skill transformation, what aspects should the government, enterprises, and higher education institutions prioritize, respectively, in optimizing music workforce development strategies?” (Flew, 2021; Ansell and Gash, 2008).

### 3.4 Data analysis

Thematic analysis served as the primary analytical method for achieving the data analysis objectives and exploring the research aims (Braun and Clarke, 2006, 2019). This research followed the concept of interpretative phenomenological methodology (Smith et al., 2009; Larkin et al., 2006) and therefore applied a six-stage thematic analysis procedure that aimed to bring out the general structural patterns without interfering with the experiential world of the research participants.

The analytical steps were carried out in a series of interrelated steps (Nowell et al., 2017; Terry et al., 2017). Familiarization included going through the transcriptions multiple times and forming a detailed comprehension of the content and written early impressions (Maguire and Delahunt, 2017). Open Coding kept the first mode of coding with the help of gerunds to identify the behavioral patterns and cognitive expressions of people in reaction to AI-related disturbances, which created open nodes (Charmaz, 2014; Saldaña, 2021). Theme Identification merged the related codes to create initial themes and define possible categories (Guest et al., 2012; Vaismoradi et al., 2016). Theme Review included case comparisons and negative case analysis to test the theme consistency and representativeness (Patton, 2015; Lincoln and Guba, 1985). Theme Refinement defined the boundaries of themes and, guided by the organizational learning and dynamic capability theories, entailed the naming of themes and interpreting mechanisms (Braun and Clarke, 2022; Miles et al., 2014). The Final Presentation presented the research findings in a three-tier model of Skill Structure Transformation–Stakeholder Response–Development Strategy, which developed a system of analysis in logic and hierarchical structure (Saldaña, 2021; Tracy, 2010).

Thematic saturation was achieved after coding transcripts from 17 participants, as no new codes or concepts emerged thereafter (Guest et al., 2006; Hennink et al., 2017). Transcripts from interviews 18 to 20 were subsequently analyzed to verify saturation, and negative case analysis was employed to enhance the robustness of the analysis (Morse, 2015; Saunders et al., 2018).

NVivo 14 software facilitated the processes of code identification, sub-theme identification, theme development, and data analysis (Jackson and Bazeley, 2019; Woolf and Silver, 2018). It was particularly suitable for this study due to its user-friendly interface, robust coding capabilities, and advanced visualization tools for identifying patterns within the dataset (Phillips and Lu, 2018; Paulus et al., 2014).

Several validation measures were used in order to ensure reliability and authenticity of the research. Member checking was done by asking the participants to confirm the viewpoints of the research based on their experiences. Researcher triangulation involved two researchers coding the data separately, negotiating the differences, and applying the final coding framework to the rest of the transcripts. Reflective documentation kept a continuous record of reflections of the researcher during this process, keeping a check of possible bias and effects of subjective positioning to avoid lack of transparency and credibility in the analysis.

### 3.5 Ethical considerations and research scope

This study adhered to strict academic ethical standards at all stages of the investigation. All participants were informed of the purpose of the study and voluntarily agreed to participate in the interviews.

Each participant was informed about the study by signing an informed consent before the interview. The information in all interviews was anonymized and the data gathered were not used commercially or otherwise in non-research contexts. The research was ethically approved and exempt by the Ethics Committee of Hanshan Normal University.

There are significant boundaries of interpretation introduced by case selection and limitations. Beijing's music industry has been chosen as the case study area due to its economic representativeness in the region and possible data availability, since it is a feasible area for preliminary theoretical mechanism exploration. The sample of the region, though, restricts the national representativeness of the results. The methodological scope utilized qualitative interviews and thematic analysis to explain the mechanisms and construct strategies, but the attribution analysis, trend prediction, and confirming music talent transformation through quantitative measures will need future studies to be extended and confirmed.

## 4 Finding

### 4.1 AI-Driven changes in skill structure and stakeholder perceptions (RQ1)

This part shows results of the first research objective: to examine the ways in which the AI technology transforms the skill base and job demands in the music industry. NVivo 14 thematic analysis revealed the importance of the identified themes regarding AI-driven job role and skills restructuring, which was presented in Table 2.

**Table 2:** Themes of job role and skill restructuring.

Theme	Sub-theme	Frequency (N)
Stages of AI application	Creation stage	15
	Production stage	18
	Distribution and promotion stage	14
	Copyright management	13
Transformation impact	Work competency restructuring and skill upgrading	19
	Cognitive transformation and learning adaptation	16
	Organizational response and institutional change	7
Stakeholder perceptions	Government awareness and policy guidance	18
	Lagging awareness in educational institutions	20
	Stratified awareness and response capability in enterprises	20
	Cognitive gaps and skill barriers among musicians	20

#### 4.1.1 AI applications across various stages of music creation

The interviewees' descriptions of AI applications in the different stages and workflows of the music industry revealed a multi-phase pattern of technological penetration across creative, production, distribution, and copyright management functions. Government officials, university scholars, corporate managers, and frontline musicians all told their stories about the systematic insertion of AI technology into the processes of traditional music.

In the creation phase, AI-assisted composition, the optimization of melody generation, and intelligent suggestions of arrangement are used. The adoption of AI essentially changes initial conceptualization and analysis potentials of the creative process.

The creation phase entails the incorporation of AI composition systems and melody optimization by means of algorithms. (E1)

The use of AI-aided organizational and smart harmony proposals is gaining growing popularity in the creation phase. (M4)

Most independent artists are currently using AI to create demos promptly and then hand-polish on that basis. (W3)

Intelligent mixing and mastering, AI-based sound design, automatic audio repair and vocal processing are all used in the production stage. This step indicates that AI transforms the conventional methods of using experience to operate audio engineering to automated cooperation based on data.

Intelligent algorithms in mixing and mastering... tools like automatic equalizers and dynamic processors are being gradually promoted. (E2)

AI can automatically identify noise in audio for denoising, and intelligently separate vocals from accompaniment. (W5)

The intelligent mixing system in our project can automatically adjust spectral balance based on reference tracks, greatly improving efficiency. (W7)

Applications in distribution and promotion involve algorithm-based recommendation systems, audience data analysis and targeted marketing, intelligent cover design, and social media content generation. These applications enhance the scientific management and responsiveness of post-release music promotion.

Intelligent recommendation in the distribution stage, such as algorithm-based recommendations on streaming platforms and targeted delivery based on user behavior data. (E1)

Like audience profiling analysis, play data monitoring, social media sentiment analysis. This data helps us better understand listener needs. (W2)

AI can also automatically generate album cover design options, even optimizing visual content for different platforms. (W8)

Applications in copyright management span comprehensive copyright monitoring and identification, intelligent royalty distribution systems, infringement detection and rights protection, and copyright tracking integrated with blockchain. These applications significantly enhance copyright management capabilities, improving real-time responsiveness, visibility, and intelligent decision-making.

AI applications in copyright management, such as audio fingerprinting technology, can quickly detect infringement. (M2)

Intelligent copyright distribution systems can precisely track the usage of each song and automatically calculate royalties. (W6)

There are also blockchain-based copyright tracking systems, making the flow of copyright more transparent and traceable. (W7)

The themes emerging from the interviewees' descriptions regarding transformation patterns and changes in talent development indicate that AI applications are evolving from relatively isolated technical interventions to systematic integration, simultaneously shifting from supporting function-based tasks and responsibilities toward the holistic restructuring of tasks and workflows. Technology embedding occurs both within specific stage-based tasks and through the reordering and new logic of management structures, organizational forms, and capabilities. The interviewees affirmatively recognize that AI makes positive contributions to efficiency, quality, and enhanced management effectiveness and work architecture; these contributions all provide the dynamic elements for talent redeployment and skill system actions.

#### 4.1.2 Observed transformations

The interviewees' descriptions of the changes brought by AI development to music job responsibilities and skill requirements revealed transformation impacts related to three interconnected dimensions: work competency restructuring and responsibility skill development; cognitive transformation and corresponding adaptation and learning mechanisms; and organizational response as institutional change. In particular, these dimensions disclose the profound structural shifts within the music talent system under the influence of AI technology, or changes in the mindset, thinking, and capabilities of the talent system.

Work Competency Restructuring and Skill Upgrading represented the most frequently mentioned transformation impact by interviewees. The tasks associated with traditional music work of playing instruments and auditory perception are moving to a system of composite competency standards, in which emphasis is placed on digital technology, data understanding and interpretation, human-computer interface, and sophisticated collaborative skills. Such transformation shows how informatization diffuses and confronts grassroots positions and establishes new capability requirements.

The key point of both government officials and academic experts were on the renewing of systemic skills structure, but corporate managers were interested in adapting skills needs on different music positions.

Positions previously oriented toward instrument performance and experience now increasingly require mastery of software operation skills, data analysis capabilities, and even basic programming. (E1)

The work skill structure is changing from single, experience-based structure to a composite, digital type. (G1)

Music producers need to understand how to analyze the data results provided by AI and optimize creative direction accordingly, technicians need to master the coordinated use of intelligent tools, and frontline musicians also need to learn to collaborate with digital platforms for work uploads or data feedback. (M3)

The frontline musician group directly experienced the rising skill threshold and reshaping of work structure through their daily work experience.

These changes alter the requirements for music directors; they need to learn how to read platform data, understand system logic, and also know how to use electronic devices for daily reporting and problem feedback. (W6)

Work boundaries have become blurred... Now I also need to learn to operate some systems and even understand basic data analysis. (W8)

The response to the varying skill demands also led to Cognitive Transformation and Learning Adaptation among musicians since the skill demands were constantly changing. The change consists of a paradigm shift in experience-based to data-driven approaches to decisions, greater sensitivity to lifelong learning and interdisciplinary competency needs, and the change in the generational gap and digital ability.

The government, academic scholars, and corporate leaders acknowledged that AI advancement compels practitioners not only to shift to decision making based on experience to the understanding of data and system integration but also requires them to deepen their knowledge in a lifetime and acquire cross-functional skills.

Traditional empiricism is being replaced by digitalization and systematization. (M1)

This places higher demands on practitioners' learning ability and lifelong growth. (G1)

This compels music practitioners to possess interdisciplinary integration capabilities and a consciousness of continuous learning. (E3)

Feedback from frontline musicians revealed generational gaps and adaptation difficulties during the digital transition, highlighting cognitive differences between seasoned and emerging musicians.

For us older musicians, although we have experience, adapting to system software is definitely slower; we must keep learning to keep up. (W2)

For young musicians like me... I'm also teaching myself some basic software operations, but I truly feel my knowledge foundation is too narrow. (W3)

This is a challenge for young people, but the pressure is even greater for older musicians; those who can't keep up risk being marginalized. (W6)

Organizational Response and Institutional Change addresses how the changes in work responsibilities and the restructuring of skill structures caused by AI are forcing deep adjustments in the music industry's vocational education system, skill certification mechanisms, and human resource management systems.

This implies that vocational education should be radically changed. (E1)

Such change gives rise to an acute necessity of the systematic renewal of the standards of the level of professional skills and system of certification of the skills. (G2)

These transformations compel us to enhance the evaluation of such areas as technical understanding and data literacy during the recruitment and training stages. (M2)

The descriptions of the interviewees show that AI technology is radically redefining the job tasks and skillset of the music business, where the approaches were rooted in instrument play and experience orientation toward the capacity system that is grounded on data-driven processes, technological composability and system collaboration. This change does not just produce work competency upgrades and role integration, but it also demands practitioners to attain profound adaptation of cognitive model and learning processes, especially in areas of considerable disparities in skill and adaptive pressures across generation lines. At the same time, AI development poses systemic questions to vacational training, skills qualification process, and talent identification that require changes in the organization of education and training, development of standards, and recruitment processes so that music talent trained in the classical training system can be transferred into digital musicians.

#### 4.1.3 Stakeholder perceptions of AI-driven talent changes

The interviewees' illustrations on how various groups, such as government, enterprises, vocational training institutions, and musicians, view AI-induced talent changes, demonstrate that there is a specific pattern of cognition and lack of coordination between the four major categories of stakeholders. These views elucidate the trends of the distribution of knowledge and the gaps in coordination systems within the music talent system with an impact of the AI technology.

Government Awareness and Policy Guidance is comparatively coherent at the level of strategic cognition and top-level design with several policies encouraging the emergence of smart music and digital culture.

We attach great importance to the intelligent development of the music industry and have already issued the 'Beijing Smart Music Industry Development Implementation Plan as a dedicated document. (G1)

The government has a clear understanding at the policy level, and we are also promoting the development of digital music and cultural construction, accelerating the intelligent transformation and upgrading of the music industry. (G2)

However, interviewees identified obstacles in the policy transmission process and insufficient awareness of policies at the grassroots level.

The government has macro-level plans for developing smart music, but the translation of policies into frontline implementation mechanisms still needs strengthening. (E4)

The government has a certain awareness of strategic deployment, such as promoting smart music demonstration projects, but gaps remain in grassroots implementation. (M4)

Lagging Awareness in Educational Institutions manifests as delayed overall institutional cognition and sluggish response, characterized by outdated course content, slow training response, and insufficient industry-education integration.

Course updates in vocational colleges and training institutions cannot keep pace with technological evolution; most still focus primarily on traditional techniques. (M3)

Training schools are slow to update their curricula. When I was in vocational school, I hadn't truly encountered content like AI music tools. It was only after entering the workforce that I discovered they were already in use, showing that education and the industry are still disconnected. (W4)

Some courses still lean toward tradition, with understanding of AI remaining at a conceptual level, lacking contextualized teaching. (W6)

Stratified Awareness and Response Capability in Enterprises reveal obvious cognitive stratification and differences in response capacity. Large enterprises generally possess awareness of digital transformation, while most small and medium-sized enterprises remain in an observational and passively responsive state.

Large enterprises have already begun strategic deployment for smart music, while most small and medium-sized enterprises are still in the exploration and observation stage. (M3)

Large enterprises have a relatively clear understanding, especially state-owned enterprises like ours, which are basically promoting systematic “smart music” development. (W6)

Cognitive Gaps and Skill Barriers among Musicians represent the widespread cognitive gaps and transformation obstacles within the musician community. Frontline musicians have a relatively vague understanding of AI, commonly existing in states of confusion, wait-and-see, and anxiety. Experienced musicians exhibit resistance to technological change, while some younger musicians show a willingness to learn proactively but lack clear pathway support.

Some people feel very uneasy upon hearing about AI, thinking they will be replaced. (W1)

We musicians tend to be accustomed to old methods, finding new things both complex and somewhat off-putting. (W2)

On one hand, I find these technologies quite novel; on the other hand, I don't know specifically how or what to learn. (W3)

Many technical topics weren't taught in school. At work, we can only rely on self-study or asking seniors. However, there were male colleagues who thought that the women were not fit for operating complicated machinery or even having a say in the AI area. It was not that women could not acquire knowledge but rather the learning platforms were not geared toward absolute beginners and the atmosphere around was not conducive for women. (W8)

The coordination challenges among stakeholder groups indicate that although the impact of AI on the music talent structure is evident at multiple levels, significant information gaps and adaptive differences persist from strategic cognition to practical response. The cognitive chain connecting the government, enterprises, educational institutions, and musicians has not yet been truly integrated, preventing policy implementation, skill transformation, and learning support mechanisms from forming an effective closed loop. This necessitates promoting multi-stakeholder collaboration to respond to the challenge of AI-driven talent restructuring.

### 4.2 Optimizing music talent development strategies (RQ2)

This section outlines the findings addressing the third research objective: to propose talent development strategies that support music talent in adapting to AI and transitioning from traditional musicians to digital musicians. Thematic analysis using NVivo 14 identified both strategic approaches and systemic deficiencies in current development practices, as detailed in Table 3.

**Table 3:** Thematic analysis using NVivo 14 identified both strategic approaches and systemic deficiencies in current development practices.

Theme	Sub-theme	Frequency (N)
Response measures	Policy and institutional-level reforms	4
	Transformation in talent cultivation content and methods	5
	Training mechanisms and organizational methods	16
Training system deficiencies	Lagging training content	14
	Institutional and resource imbalances	17
	Insufficient job alignment	18
	Rigid teaching methods	16
	Lack of evaluation and incentives	16
Development strategy optimization	Institutional support and resource guarantees	19
	Educational reform and capacity building	20
	Enterprise training and job role transformation	20
	Communal support and growth pathways	10

#### 4.2.1 Organizational measures in response to skill changes

The illustrations of organizational actions provided by the interviewees when responding to the change in skills details that it is systematic and can be encapsulated into three interconnected dimensions, namely, reforms on the policy and institutional level, change in the content and methods of talent cultivation, and the reorganization of training mechanisms and organizational practices. These replies illustrate how the music industry is acting as a collective response toward the problem of AI induced change in talent skill sets.

Transformations at the Policy and Institutional Levels suggest that government agencies are vigorously pushing the changes in skill systems and classification of occupations, and their main concern is the creation of a new institutional framework that suits the intelligent music environment.

Collaborating with relevant departments to formulate job standards and talent evaluation indicator systems for Intelligent Music Technicians. (G1)

Including new occupations such as Music Technology System Technicians into the updated directory. (G2)

Organizing pilot projects for professional skill level certification to promote the integration of academic certificates + skill level certificates (dual-certificate integration). (G3)

Transformation in Talent Cultivation Content and Methods demonstrates how training institutions implement multi-dimensional initiatives through curriculum system reform, practical base construction, and industry-education integration to enhance music talents' adaptability to AI applications. These changes help optimize the talent supply structure from the source and narrow the gap between education and job requirements.

Our college has recently added content on intelligent music, music informatization, and AI technology to its curriculum system, while also promoting the construction of joint industry-university training bases and introducing project-based case teaching. (E2)

The college I belong to has made active attempts in curriculum system reform, integrating modules such as intelligent music, data-driven creation, and music production software application, and promoting the integration of practical teaching and project-based teaching. (E4)

Training Mechanisms and Organizational Methods reveal a strong practice-oriented and job-adaptive approach at the corporate level. Organizations implement methods such as project-driven learning, guidance from experienced musicians, and learning by doing to achieve on-site skill transformation.

Our company primarily promotes technological upgrading through project practice. For example, by introducing AI-assisted systems in some projects and arranging training, where project managers lead frontline musicians in learning. (M3)

Sometimes the project department lets us learn while working. For example, arranging a team leader to learn the system operation first, then teaching everyone step-by-step. (W2)

Online learning platforms and digital learning resources are gradually permeating, achieving an organic integration of spatiotemporal flexibility and content diversity.

The company also has an internal online course platform dedicated to providing learning videos about music production software and intelligent music. (W4)

The descriptions indicate that organizations in the music industry are actively responding to the pressure of AI-driven skill transformation through a triple path of institution-driven initiatives, content reform, and mechanism innovation. However, interview evidence reveals differences in the degree of implementation across different organizations and groups, presenting a pattern of “leading organizations driving, mid-level responding, grass roots diverging.” Future development needs to strengthen the institution-practice linkage mechanism to promote the widespread permeation of standards, resources, and capabilities within organizations, achieving deep structural transformation and comprehensive adaptation of the music talent structure.

#### 4.2.2 Current deficiencies in the training system

The opinions of the interviewees regarding gaps in the present music training system to address the skills transformation caused by AI demonstrate systematic breakpoints in five dimensions connected to each other. These loopholes restrict the dynamism of the training platform and reveal the organizational failures of the AI-led ecosystem.

Another general agreement between interviewees became Lagging Training Content. The existing training programs remain based on traditional methods of music, with no topics about new technologies and as such do not contribute to the growth of digital musicians.

There is lack of involvement between content; the curriculum system is not able to match the latest technological advancement such as intelligent music, AI, and data analysis. (M3)

The training system is made to prioritize the traditional methods and the non-dynamic knowledge without developing the dynamic capabilities to make intelligent music. (M5)

The quality of training is highly of the institutional and resource imbalances. Centralized distribution of resources, imbalanced geographical coverage, and ineffective mechanisms of the industry-education cooperation that lead to the lack of training opportunities and support of independent musicians and grassroots music workers were identified by interviewees.

Most existing resources are concentrated on technical management personnel, while training coverage for grassroots music creators is very low. (M3)

There aren't many training opportunities, especially for independent musicians. Even if we want to pursue further training, there's nowhere to go. (W1)

Most equipment is typically used by management. We musicians don't have access to it; there's no opportunity to practice even if we want to. (W3)

Insufficient flexibility in training schedules exacerbates implementation difficulties.

The format isn't flexible enough. Many training courses are scheduled at fixed times, conflicting with creative schedules. (W7)

Many technical topics weren't taught in school. At work, we can only rely on self-study or asking seniors. However, there were male colleagues who thought that the women were not fit for operating complicated machinery or even having a say in the AI area. It was not that women could not acquire knowledge but rather the learning platforms were not geared toward absolute beginners and the atmosphere around was not conducive for women. (W8)

Gender and experience barriers further restrict access to learning resources.

Many technical topics weren't taught in school. At work, we can only rely on self-study or asking seniors. However, there were male colleagues who thought that the women were not fit for operating complicated machinery or even having a say in the AI area. It was not that women could not acquire knowledge but rather the learning platforms were not geared toward absolute beginners and the atmosphere around was not conducive for women. (W8)

Insufficient Job Alignment stands out as the major barrier to the effective use of training. The training content has no specificity, it does not provide classification and stratification according to job level, professional category, and learning foundation; as a result, the outcomes are impractical, hard to remember, and difficult to transfer.

The biggest issue is the unclear hierarchy and lack of targeting. Let's say for instance that novice musicians like us need purely and practically introductory training and not the most advanced features right from the start. (W3)

A lot of the courses are designed to be the same for everyone, putting young and old students, technical staff, and general musicians all together. This is an inefficient approach that cannot provide personalized guidance. What we require is specialized practical training according to our different categories. (W4)

Rigid Teaching Methods are a factor that has a direct effect on the training experience. Presently, the training is heavily dependent on PowerPoint slides, which do not allow for immersive, hands-on, and project-based teaching methods. The musicians cannot acquire the skills through the real scenarios, this further engages and reduces the overall effectiveness of learning.

There is no coordination with the actual operational scenarios. The training methods are too rigid, only PowerPoint presentations without the opportunity to truly interact with the equipment. (W2)

Some new technology training still remains at the level of PowerPoint explanations, without live demonstrations or virtual operation environments. (W8)

Absence of Evaluation and Incentives weakens intrinsic learning motivation. The training system lacks a scientific effectiveness evaluation system and promotion incentive mechanisms. The results of the training do not provide significant returns, and as a consequence, the frontline musicians become less and less willing to improve their skills.

The career progress paths are not linked, and this causes the trained musicians to perceive no value in promotions and salaries, thus affecting their enthusiasm for training and its sustainability. (G2)

There is no continuous training nor any incentive schemes. It's unclear whether the learning is useful or whether performing well leads to promotion opportunities. (W3)

The interviewees' descriptions indicate that the current training system in the music industry has not yet established a supply logic and structural mechanism deeply aligned with the AI environment. It covers everything from content to methods, from institutions to incentives, and reveals a disconnection from the demands of digital transformation. A systematic reconstruction and supply-side upgrade of the training system, with task orientation, competency-based approaches, and job alignment as its core logic, is urgently needed to effectively support the structural transformation of music talent.

#### 4.2.3 Optimizing music talent development strategies

The interviewees' views on how the government, enterprises, and training institutions should respectively optimize music talent development strategies to address the skill transformation brought by AI reveal directions for collaborative governance and key mechanism gaps across four strategic dimensions. These dimensions clarify the coordination requirements within the music talent system under the influence of AI technology.

The very first basis to develop a smart music talent ecosystem is Institutional Support and Resource Guarantees. The government plays a leading role in top institutional design and financial backing devoted to the establishment of smart music vocational skill standards, certification systems, and comprehensive education and training infrastructure; this not only leads to the elevation of talent development in the whole country but also provides the policy ground for distribution of resources.

The government should formulate a special plan for AI talent development in the music industry, introduce a digital musician cultivation policy, establish a special fund to support vocational colleges in offering smart music-related majors, and promote the establishment of a smart music vocational education standard system. (E1)

The government should lead the construction of the policy system and standard framework for smart music talent development, set up special funds to support regional training bases and course content R&D, and promote the establishment of a certification system covering the entire career chain. (G1)

Education reform and capacity building emerge as the main goal to transform talent structures. Higher education institutions must keep pace with the evolution of AI and smart music technology, reconstruct curricula by introducing real engineering cases, and drive the shift from theoretical teaching to project-driven and scenario-based practical training. Teaching methodologies' advancement and further alignment of industry partnerships enhance effectiveness in training and experiential learning.

Training institutions and universities should proactively reform their curriculum systems, introduce practical AI cases from the processes of music creation, production, and distribution, promote the synchronous updating of teaching content with technology, improve training precision and effectiveness, and jointly advance the enhancement of music talent toward digitalization and intelligence. (M3)

Training institutions need to step out of the classroom and into recording studios and live performance venues, designing targeted practical courses according to different specializations so we can truly learn and apply. (W2)

Enterprise Training and Job Role Transformation serves as the executive hub driving talent structure transformation. The willingness of employees to participate in technological growth and have the motivation for career advancement can be increased by organizations setting up job-related training measures, specifying the internal job switching routes and skills development paths, and then linking training incentives to the performance mechanisms.

Enterprises should make training routine and structured. Everyone from executives to frontline musicians should have a clear skill transformation pathway, and training outcomes should be incorporated into assessment and promotion mechanisms. (M4)

Companies can implement refined management of training through stratification and classification. For example, by establishing a Young Musician Growth Plan that allows novices like us to master skills in stages and have a promotion channel. (W4)

Enterprises should establish a job-skill-incentive' linkage mechanism. As a specific example, it could be that the musicians studying drone operation or scheduling systems would get technical allowances or be promoted by stars. (W5)

Communal Support and Growth Pathways express a very strong concern for the diversity of talents plus their different developmental stages. The people being interviewed suggested creating more precise training paths according to job levels, professional attributes, age structure, and gender differences, thus making a growth loop from entry to mastery and from training to promotion that would lead to employee capability transformation.

We cannot keep on training people with the approach of “letting the old ones adapt to the new tools.” On the contrary, we should be reshaping the growth path for a new generation of “smart music talent.”

Overall, talent development needs to be upgraded from “supplementing skills” to “building an ecosystem,” achieving a comprehensive transformation from “traditional musicians” to “digital musicians.”

Special attention to young musicians, women, and career changers supports their transition to digital musicians.

Enterprises should incorporate smart device training into the new employee onboarding process and specifically establish “young technical talent promotion channels.” (W3)

Establish special classes for female music talent to encourage more people to transition from supporting workers to smart music practitioners. (W7)

The perspectives of the interviewees indicate that in order to cope with the issue of the transformation of talented musicians by AI technology, it is necessary to overcome the fragmentation and slowness of the old training methods and to effectively synchronize the government, industry, and academic sectors.

An elaborate, multi-dimensional, and gradual human resource development framework can be formed via the four-pillar strategy of institutional support, progressive education, corporate involvement, and social upliftment. This framework is made up of various stakeholders, keeps up with the latest tech trends, and facilitates the music industry's shift in structure from conventional to digitally skilled musicians.

## 5 Discussion

### 5.1 Skill restructuring under AI and stakeholder perceptions

The AI technology has also been integrated into the fundamental functions of the music industries, i.e., creation, production, distribution, and copyright management, which are fundamentally transforming the skill structure of the industry (Leyshon, 2009). This change is not an isolated trend in the creative industries but a shift toward augmentation, as opposed to replacement, of human ability, and toward composite competency systems based on digital tools, interoperability, and cross-disciplinary integration (Briot et al., 2020; Acemoglu and Restrepo, 2022). This means that music jobs are beginning to take on the same boundaryless work characteristics and shared performance expectations, which are reminiscent of recent empirical results of polarized skills and task restructuring in response to AI-driven technological change (Acemoglu and Restrepo, 2022).

The identified transformation pathways in this study imply cognitive transformation, transitioning to tool adaptation and finally to task integration. Frontline musicians are moving on to being platform collaborators, as well as project managers becoming data-driven decision-makers. Such a loss of responsibilities is in line with the fact that AI technologies erode traditional occupational segregations and exert pressure to develop general adaptive strengths across job and system (Bessen et al., 2020).

The power of AI is not only in the peripheral automation, but it also helps to restructure the managerial support function, as well as the frontline operational positions in music production systems. Instead of job replacement, AI enables new work forms based on system coordination and human-algorithm interaction. Empirical research shows that the scheduling, copyright enforcement, and creative workflow management domain is more and more based on the jointness of human and AI decisions, which points to a new standard paradigm of role restructuring (Raisch and Krakowski, 2021).

In line with this, AI-based reconstruction of music roles focuses on composite competencies that involve collaborative skills, system knowledge, and judgment skills. This transition is consistent with dynamic capability theory that values the significance of organization learning and reconfiguration of capabilities to tackle technological uncertainty (Teece, 2018; Eisenhardt et al., 2010). These lessons offer theoretical foundations for job redesign and education redesign in AI-enhanced creative sectors.

Multi-stakeholder cognitive stratification and lack of consistent response capacities further manifest through the creation of structural disconnections between policy intent, organizational practice, and individual behavior as an outcome of AI-empowered development of music talents (Sotiriadou et al., 2014). Although there are detailed policy designs in digital skill development, there are gaps in the implementation because of the lack of institutional penetration and vertical transmission systems, a trend common in skills policy studies (Organisation for Economic Co-operation and Development, 2021).

The responses of corporations are characterized by evident stratification: large companies use their resources more effectively to develop AI pilot systems and integrated training paths, whereas small and medium enterprises are not able to overcome financial and organizational constraints. This kind of asymmetry indicates differences in resource-based adaptive capacity and supports the inequality in outcome of talent transformation (Bessen et al., 2020).

Institutions of learning are not keeping pace with occupational transformation, and have archaic programs, minimal AI education, and impoverished practice training infrastructures, which make skills mismatches worse. The results align with the observations that vocational and higher education systems are slow in adapting to the requirements of digital transformation (Bond et al., 2021; Collins et al., 2003).

At the individual level, musicians experience generational differences in cognition and emotional opposition. Anxiety and rejection of AI systems by older musicians is common, and even younger musicians, although more accepting, are not provided with structured support by organizations to translate willingness to learn into transformation ability. These differences in behavioral patterns complement the Technology Acceptance Model by illustrating the mediating power of institutional support and competency perception in determining AI-based learning behavior (Venkatesh et al., 2020).

In general, the results indicate that the introduction of AI into the music industries is bound more by the lack of coordination through various levels of institutional, organizational, educational, and individual than technological inability. To overcome these problems, it is necessary to implement more diffuse policy transmission, supply education system flexibility, and coherent organizational support systems to transition to systems coordination rather than structural fragmentation.

### 5.2 Moving toward an ecosystem approach to talent development

To fully address the multi-level coordination challenges, it is necessary to focus on integrating learning and training models and moving toward comprehensive ecosystem-wide development, rather than concentrating on fragmented or hierarchical training levels. The detected skills gaps, cognitive differences, and disjointed institutional actions require a comprehensive model comprising four pillars such as institutional support, educational reform, enterprise engagement, and community development pathways to support the process of transforming traditional musicians into digital musicians. This ecosystem solution aligns with the framework of lifelong learning and multi-stakeholder collaboration characteristic of the OECD, which focuses on coordinated governance, skills recognition, and constant creation of capabilities across industries (Organisation for Economic Co-operation and Development, 2021), and well as with the orientation of Chinese policy toward industry-education integration and government-enterprise collaboration (Li and Zhang, 2022; Kong, 2014).

The institutional infrastructure is developed as the basis for dealing with the shortcomings in cross-level coordination and job-matching orientation in the current policy systems. According to the empirical investigations, it is observed that fragmented policy tools usually cause poor transmission effect and low structural influence on the transformation of the workforce (Organisation for Economic Co-operation and Development, 2021). An institutional guarantee loop consisting of skills standard systems, certification systems, and specific fiscal subsidies can be an immense boost to the structural penetration of transformation strategies. This rationale fits recent research on policy integration instruments, which emphasize the highest-level planning, capability to address the needs of relevant implementation, and efficient mobilization of its resources regarding technology-based labor transitions (Teece, 2018).

The change of the educational system is a reaction to the uncovered gaps of backward content of the curriculum, a single pedagogical pattern, and unequal distribution of resources in the regions, which weaken the process of developing composite competencies to be used in the creative industries of the AI era. Studies on the digital transformation of higher education imply that the modernization of the curriculum, pedagogical innovation, and practice-based platforms should develop in a synchronized way to transform education into competency-creating instead of knowledge-delivering (Bond et al., 2021). In the case of music education, this involves the use of AI-based composition, software, and data analytics modules, virtual simulation, and project-based learning methods, to improve experiential learning and professional flexibility (Briot et al., 2020).

The gaps in systematic job training opportunities and effective systems of skill incentives and benefits that frequently lead to musicians being willing to learn but incapable of carrying out career shifts are overcome through enterprise capacity building. Enterprise strategic human resource development needs to put in place skill improvement channels in line with job chains such as job transition re-education, tiered certification, and performance-based rewards. This strategy is similar to the dynamic capabilities viewpoint, which emphasizes the importance of organizational learning and reconfiguring capabilities as the key reaction to technological uncertainty (Teece, 2018).

An inclusive community support system further responds to the structural disadvantages faced by young musicians, women, and career-transitioning musicians due to insufficient adaptive institutional support. Empirical studies on technology adoption and training behavior indicate that contextual support and institutional facilitation significantly influence individuals' willingness to engage in learning and skill upgrading (Venkatesh et al., 2020). Establishing differentiated learning pathways such as women-focused digital music programs, youth-oriented technology tracks, and transition-oriented training camps can effectively enhance motivation and sustain long-term professional growth.

**Figure 1:**
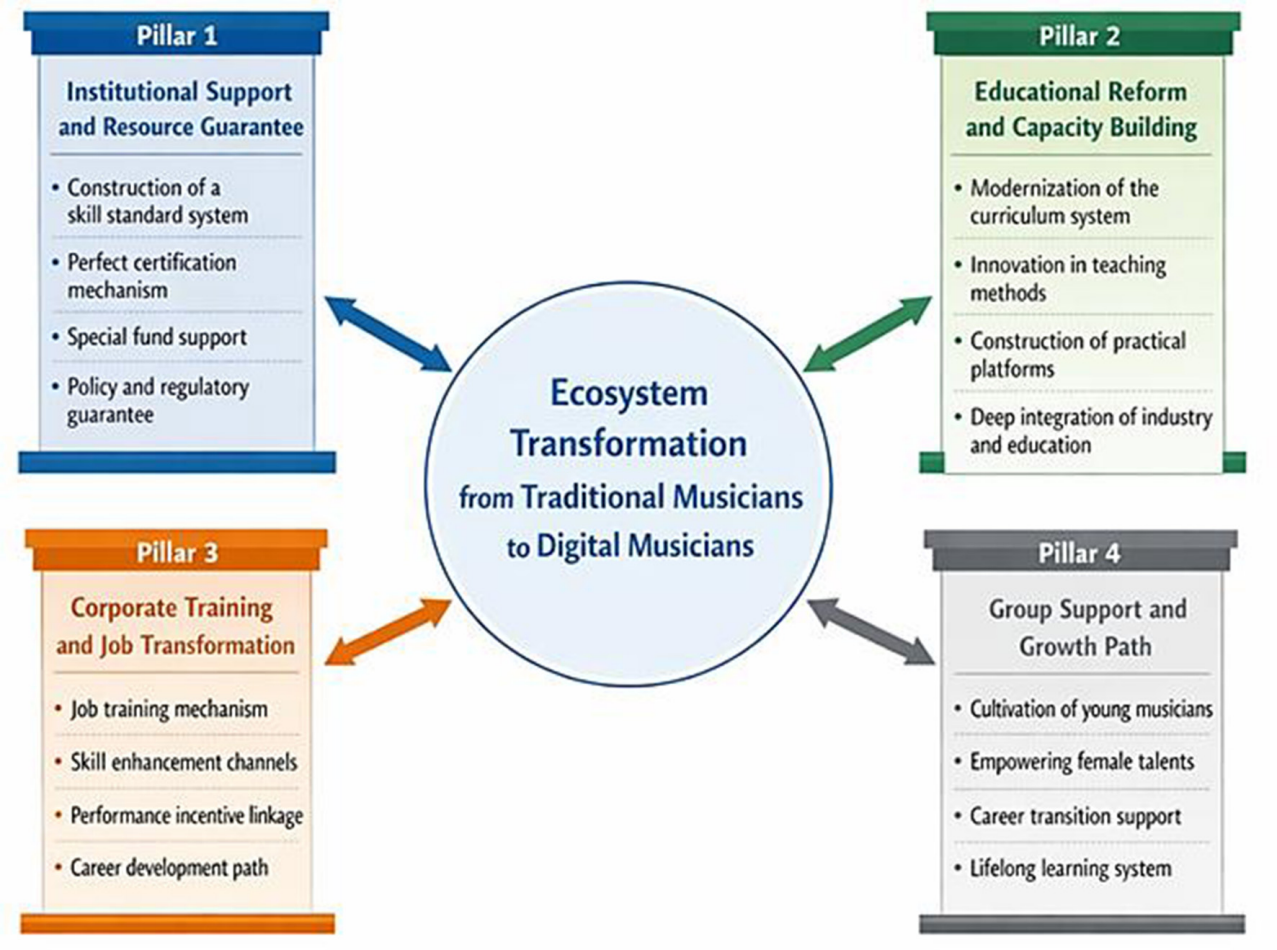
Strategic framework for talent development in the music industry.

An important point that can be drawn out of this analysis is that training must go beyond assisting current musicians to be familiar with new tools and instead redefine long-term developmental trajectories of future digital music artists. This transformation between the deficiency-based training to the ecosystem-based one is a manifestation of the essence of the exploratory organizational learning, where individuals and institutions co-create adaptive capability systems as opposed to the linear skills upgrading. To this end, the present research paper puts forward a four-pillar model of talent development, including institutional support, educational change, enterprise involvement, and community development to contribute to sustainable change in the AI-driven music industries (see Figure 1).

### 5.3 Research significance

This research investigates ways of transformation of music talent under the impact of AI and multi-stakeholder coordination of the processes, taking into account the issue of transformation of traditional musicians into digital musicians with theoretical, practical, and methodological implications. Current studies have shown that AI-based digital transformation reorganizes occupational sets of skills and requires systemic levels of coordination across institutional, organizational, and individual levels, instead of linear capacity building of skills (Raisch and Krakowski, 2021; Organisation for Economic Co-operation and Development, 2021).

The theoretical value of this research exceeds the linear AI-technology-driven paradigm through incorporating dynamic capability theory, perspectives on organizational learning, and skills ecosystem framework. Previous research points to the fact that dynamic capabilities can help organizations and individuals to sense, seize, and reconfigure competency in response to technological disruption (Teece et al., 2020), whereas theories of organizational learning remind about adaptive cognition and reconstruction of knowledge in an uncertain scenario (Argote and Miron-Spektor, 2019). The synthesis of these points of view forms the multidimensional analytical structure of job transformation-cognitive response-coordination strategy in the present research, which systematically demonstrates the structural processes of the AI-induced music talent transformation and adaptive relations between various participants. This framework will be helpful in enhancing academic knowledge on the topic of skill reconfiguration and institutional change in the digital transformation environment (Organisation for Economic Co-operation and Development, 2021).

The practical implications of this study lie in in-depth interviews of practitioners in the music industry in Beijing that demonstrate the existence of problems related to cognitive divergence, organizational responses taking too long, and the fragmentation of the music industry institutions in the context of developing music talents. These results are in line with empirical evidence that hypothesizes that ecosystem-level coordination problems frequently limit the efficiency of digital skill transformation policies (Brown et al., 2020). To this end, this research contends that a four-pillar coordination approach that includes institutional support, educational reform, enterprise engagement, and community development would be beneficial. This approach, in contrast to the linear train models, focuses on the simultaneous transformation in cognition, institutions, and capabilities, providing practical policy and practice opportunities to stakeholders in the music industries in the region.

Methodologically, the research integrates a phenomenological qualitative research design that uses a six-step thematic analysis process to achieve both analytical rigor and interpretive depth. Thematic analysis is now more often known as a versatile and orderly approach to discovering patterns among narratives of stakeholders in the complex situations of transformation (Braun and Clarke, 2021; Leech and Onwuegbuzie, 2011). This study builds a multi-level, cross-stakeholder thematic network, by open coding and sequential theme development, which enhances the methodological frameworks on talent transformation research under technological change and offers replicable advice in conducting qualitative research in creative and cultural industries in the future.

### 5.4 Limitations and future research directions

Despite the fact that this research offers an initial discussion of the pathways of music talent transformation and multi-stakeholder collaboration mechanisms under the influence of AI, there still are several limitations that need to be expanded in further research. Just like the previous qualitative research in the creative sectors, the contextuality can limit the externalization of the results to any future outward environment, which includes the local context of the research (Braun and Clarke, 2021).

Foremost, in Beijing the sample is intense. Even though there are various respondents, including government officials, enterprise managers, educational instructors, and frontline musicians, geographical distribution, the size of enterprises, and job hierarchy can lead to the limitation of the external validity of the findings. The current literature indicates that regional industrial organization and resources play a significant part in influencing the results of digital skill transformation, especially of small and medium-sized enterprises and of peripheral labor markets (Organisation for Economic Co-operation and Development, 2021). Further researches may expand the frames of sampling to incorporate musicians living in the countryside, in cities of various levels, and in other categories of professions to create a more accurate depiction of the transformation of musical talent.

Second, this study relies primarily on qualitative interviews to examine stakeholder perceptions and interaction mechanisms. Although the qualitative methodology works well in revealing cognitive processes and institutional dynamics in complex change situations, it is not as explanatory as quantitative validation (Creswell and Plano Clark, 2018). Future studies may follow mixed-method designs including surveys, structural equation modeling, or system dynamics modeling to investigate the causal relationships between training investment, cognitive disparity, and job-role fit to enhance the rigor of the theoretical constructs (Venkatesh et al., 2020).

Third, this study mainly captures the static status quo of music talent development and pays limited attention to the dynamic adaptation mechanisms accompanying the continuous evolution of AI technologies. According to previous studies, digital transformation by nature is a long-term process, which is marked by learning feedback loops, path dependency, and continuous capability upgrading (Teece et al., 2020). Longitudinal designs, process-tracing designs, or comparative time series analysis would be more effective in showing the co-evolution of policy changes, job restructuring, and adaptation of the musicians over time.

Lastly, even though there is a variety of stakeholder views, too little attention is paid to the unique adaptation obstacles encountered by disadvantaged groups: women, young performers, and career-shifting people. According to recent research, various forms of institutional support, identity formation, and the encouragement of underrepresented populations should be differentiated when it comes to inclusive digital transformation (International Labour Organization, 2022). The evolution of career identity, changes in technology perception, and empowerment processes of these groups in the future should be systematically studied to inform development in AI-driven creative sector talent development strategies, including everyone.

## 6 Conclusion

This study takes from traditional musicians to digital musicians as its core theme, focusing on the AI technology-driven transformation of music talent structure. It methodically answers two major questions: how AI is redefining job positions and skillsets within the music business; how various parties vary in their perception and reactions to talent change; and how talent strategies can be created to help the music industry persistently adjust and offer talent updating through the AI period.

The research on 20 interviewees in Beijing shows that the whole process of creating, producing, distributing, and administering copyrights has been largely transformed into the usage of AI technology and that occupations have shifted toward data-driven and system-operation orientations and that the occupation structure has been quickly moving toward more complexity and digitalization. At the same time, strategic alienation among stakeholders in cognitive awareness and responsive behavior, fragmented schemes are of a characteristic nature: government efforts are made, the level of enterprise response differs, educational institutions respond slowly, and musicians are a wait-and-see people. Frontline musicians face various challenges, such as deficient skill base, time management and psychological stress, and the available training efforts are typically faced with old content, restrictive format and lack of motivation. In reaction to this, this research paper suggests a cooperative talent development program with institutional support, education reform, enterprise involvement, and community building to develop the strength and inclusivity of the labor force system and advance the sustainable growth of the music industries.

Theoretically, this research coordinates perspectives of dynamic capabilities, organizational learning, and skills ecosystem to create an analytical model of job transformation-cognitive response-collaborative strategy, which would enhance the insight into the mechanisms underlying the transforming of the labor force by AI in the music industries and how talent development can be used to achieve the UN Sustainable Development Goals. In practice, it maps the major issues of talent development in the music industry by interviewing multi-stakeholders and uses a four-pillar collaborative approach. In the methodological approach, using a phenomenology-focused approach and thematic analysis procedure, it develops a multi-level thematic model, deepening the application paradigm of qualitative research in the context of complex technological change.

Despite the structural information about the labor force transformation processes in the context of AI presented in this study through the lenses of the multi-stakeholder collaboration, there are still some limitations, including the regional focus of the sample, one methodological approach, and the lack of dynamic processes. Further developments would be possible in the future by expanding the scope of the sample, pursuing mixed methods, enhancing the long-term process tracing, and emphasizing the mechanisms of adapting the marginalized groups. Such initiatives can help create a robust, inclusive, and sustainable music talent developing system that can offer more generalized and prospective theoretical coverage to developing nations with the challenges of intelligent transformation.

## Data Availability

The datasets presented in this article are not readily available. Confidential and ethical restrictions apply to protect participant privacy. Data contain identifiable information and were not consented for sharing. Requests to access the datasets should be directed to 20250069@hstc.edu.cn.
